# Real-world graft utilization after CTN-1101: a registry-based analysis of haploidentical graft versus umbilical cord blood trends

**DOI:** 10.1038/s41409-025-02694-z

**Published:** 2025-08-18

**Authors:** Danh T. Tran, Ruyun Jin, Hong Zhu, Gabrielle Schmidt, Stephen R. Spellman, Karen K. Ballen

**Affiliations:** 1 Department of Medicine, University of Virginia Medical Center, Charlottesville, VA USA; 2 Department of Public Health Sciences, University of Virginia School of Medicine, Charlottesville, VA USA; 3 CIBMTR (Center for International Blood & Marrow Transplant Research), NMDP, Minneapolis, MN USA; 4 University of Virginia Comprehensive Cancer Center, Charlottesville, VA USA; 5 Present Address: Fred Hutchinson Cancer Center/University of Washington, Seattle, WA USA

**Keywords:** Stem-cell research, Haematopoietic stem cells

## Abstract

Randomized clinical trials are expensive and not always practice changing. The Blood and Marrow Transplant Clinical Trials Network (CTN) 1101 trial (2012–2018) showed a lower two-year overall survival after umbilical cord blood (UCB) compared to haploidentical graft (haplo) transplants. To quantify the change in graft utilization after the trial’s publication, a cohort of 11,190 U.S. adult HCT recipients selected with inclusion/exclusion criteria similar to CTN-1101’s were analyzed across three time periods: 2010–2012 (pre-study), 2013–2018 (during-study), and 2019–2022 (post-study). We found a significant increase in haplo utilization compared to UCB, with the trend beginning around 2013. Compared to non-Hispanic White, Black recipients were more likely to receive haplo, Asian recipients were less likely, and Hispanic recipients had similar rates. We also expanded our analyses to 61,465 patients to assess haplo and UCB utilization compared to other allogeneic donors. In this cohort, utilization of alternative donor grafts increased when compared to HLA-matched related or unrelated donor grafts for Black, Hispanic, and Asian recipients. Our findings demonstrate practice change toward haplo transplants had begun before the CTN-1101 trial’s publication and continued to significantly increase afterward. HLA-mismatched donors are vital alternative graft sources, allowing patients of all backgrounds to receive HCT.

## Introduction

Allogeneic hematopoietic cell transplantation (HCT) is an important curative treatment option for malignant and non-malignant blood diseases [1]. Randomized clinical trials are considered the “gold standard” in clinical practice in oncology, including HCT, but they can require significant investment [2], without an apparent change in decision making and clinical practice.

Umbilical cord blood (UCB) and haploidentical grafts (haplo) are important donor sources for patients who do not have an optimal matched related or unrelated donor. Two parallel Phase 2 studies showed comparable one-year overall survival outcomes [3]. The US multicenter phase-3 trial Blood and Marrow Transplant Clinical Trials Network (CTN) 1101 was performed between 2012 and 2018 to directly compare outcomes of double unrelated UCB and haploidentical bone marrow (haplo-BM) transplantation for adults with hematologic malignancies receiving reduced-intensity conditioning [4]. This trial indicated no significant difference in two-year progression-free survival, but haplo-BM had better two-year overall survival compared to double unrelated UCB (57% vs 46%; *p* = 0.04). A follow-up study with longer follow-up of trial and non-trial patients confirmed the conclusions favoring haplo-BM over double UCB [5]. In this analysis, five-year survival was higher after non-trial haplo-BM compared with trial double UCB (47% versus 36%; *p* = 0.012).

Since the publication of the CTN-1101 trial and its follow-up studies, there has been no formal assessment of the impact of the findings on haplo versus UCB utilization. In the most recent report from the Center for International Blood and Marrow Transplant Research (CIBMTR), there was an increase in haplo transplants and a decrease in UCB transplants between 2009 and 2019, but the analysis was generalized to a wide age range, including pediatric patients [6]. Publication and promotion of changes made based on clinical trial data lend support for resource intensive phase 3 trials in the future. Therefore, in this study, we quantify the change in graft utilization, specifically haplo-BM and haplo-peripheral blood stem cells (haplo-PBSC) versus UCB, that occurred in real-world clinical practice after the publication of the CTN-1101 trial. We also assess the increase in all graft sources that had led to improved access to transplant.

## Materials and methods

### Database and study patients

Data was provided from the CIBMTR, which collects detailed information on all consecutive HCTs performed at U.S. Transplant Centers. All participants in the CIBMTR database provided informed consent in compliance with the Declaration of Helsinki. The study was approved by the NMDP Institutional Review Board. For this retrospective analysis, the CIBMTR provided transplant data from 246 U.S. centers on HCT performed between 2010 and 2022.

In our primary analysis, the inclusion criteria were adopted from the CTN-1101 trial [4, 5], including HCT in US adult recipients between 18 and 70 years of age who received UCB transplantation (including single and double UCB) or haplo transplantation (including both haplo-BM and haplo-PBSC) for leukemia, lymphoma, myelodysplastic syndrome (MDS), and myeloproliferative neoplasm (MPN). Non-US patients were not included since they did not participate in the CTN-1101 trial. Transplants for solid tumors and non-malignant disorders were excluded. Multiple grafts, such as UCB and BM, or PBSC and BM, or PBSC and UCB were also excluded. With these criteria, we refer to this study population as the Main Cohort (*n* = 11,190).

In our expanded analysis, in addition to the Primary Cohort, we also included recipients with age and transplant indications as above who received single BM or PBSC grafts from Human Leukocyte Antigen (HLA)-identical siblings and HLA-matched other relatives (collectively MRD), HLA-matched unrelated donors (MUD), and HLA-mismatched unrelated donors (mMUD). We refer to this study population as the Extended Cohort (*n* = 61,465).

### Study outcomes

Using the Main Cohort, the primary outcome of the study was the temporal trend demonstrating the utilization of haplo-BM and haplo-PBSC vs single and double UCB over time. Secondary outcomes were to evaluate whether demographic factors such as age, sex, race/ethnicity had any association with graft utilization and whether these factors were associated with the rate of change over time. Specifically, the likelihood of receiving haplo vs UCB in male vs female, younger (<50 years) vs older (≥50 years) patients, and Hispanic, Asian, non-Hispanic Black vs non-Hispanic White (NHW) patients was analyzed. Predicted percentage of haplo for each year was also calculated for these demographic factors using multivariable logistic regression.

In our expanded analysis using the Extended Cohort, we evaluated the utilization of haplo and UCB and other allogeneic donors, including MRD, MUD, and mMRD, and the association of graft source with the aforementioned demographic factors.

### Statistical analysis

Continuous variables were presented as “median (interquartile range, IQR)” and compared by the Wilcoxon Rank Sum test when normality assumptions were not satisfied. Categorical variables were presented as “number (percentage)” and compared using the Chi-square test between groups. Univariable and multivariable logistic regression analyses were performed to assess the association between transplant year, patient characteristics and the receipt of haplo vs UCB transplants in the Main Cohort. Transplant time was evaluated as continuous data (year) or by time periods. Interactions between patient characteristics and the time of transplant were examined to assess differences in the change of graft utilization over time between patient groups. Odds ratio (OR) with 95% confidence interval (CI) were used to represent the results. Multinomial logistic regression analyses were conducted to assess the utilization of haplo, UCB, MRD, mMUD, or MUD, adjusted by year of transplant, sex, age, and race/ethnicity, in the Extended Cohort. The Relative Risk Ratios (RRR) with 95% CI were used to represent the results. Statistical analyses were performed using R 4.2.2 software (R Foundation for Statistical Computing, Vienna, Austria).

## Results

### Primary analysis with the main cohort (11,190 patients)

#### Patient selection

**Fig. 1:**
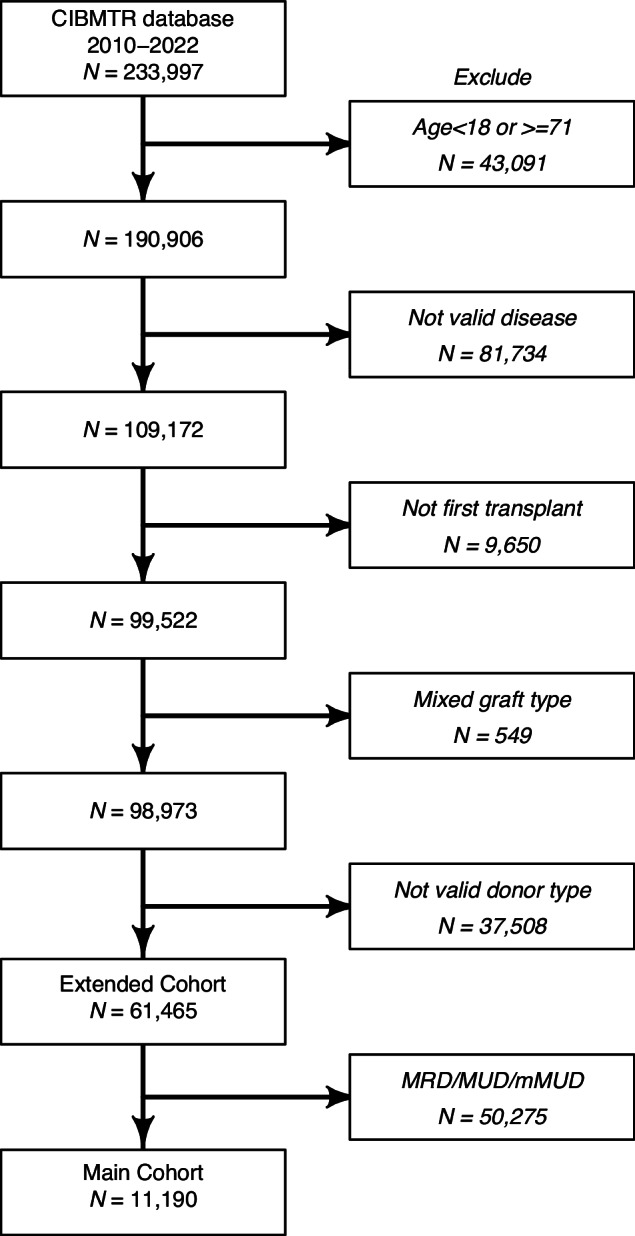
Flow chart of patient selection. Main cohort: adult recipients between 18 and 70 years of age who received the first transplant with single or double umbilical cord blood (UCB), or haploidentical bone marrow (haplo-BM) or haploidentical peripheral blood stem cells (haplo-PBSC), for leukemia, lymphoma, myelodysplastic syndrome, and myeloproliferative neoplasm. Extended cohort: criteria for Main Cohort plus HLA-identical sibling and HLA-matched other relatives (MRD), HLA-matched unrelated donor (MUD), or HLA-mismatched unrelated donor (mMUD). Both cohorts exclude transplants for solid tumors and non-malignant disorders, as well as multiple grafts, such as UCB and BM, or PBSC and BM, or PBSC and UCB.

Transplant activity data for 233,997 patients were provided by the CIBMTR. From this cohort, we excluded 43,091 patients who did not meet inclusion criteria for age, 81,734 patients who did not undergo transplants for the diseases included in the study criteria, 9650 patients who underwent re-transplants, 549 patients who had combined grafts, and 87,783 patients whose grafts were neither haplo nor UCB. After these exclusions, we identified and analyzed a cohort of 11,190 patients that closely aligns with CTN-1101’s design (Fig. 1).

#### Trend in utilization of haploidentical grafts versus umbilical cord blood

Among the Main Cohort of 11,190 patients, 8215 patients (73.4%) received haplo transplants, and 2975 patients (26.6%) received UCB transplants (Table 1A). Based on the timeline of the CTN-1101 trial with patient enrollment conducted between 2012 and 2018, we categorized these transplants into three time periods: pre-study (2010–2012), during-study (2013–2018), and post-study (2019–2022). We found over the three time periods, there was an increasing proportion of haplo vs UCB (average number of haplo transplants per year: pre-study: 153, during-study: 561, and post-study: 1098; 32%, 68% and 91% haplo out of combined haplo and UCB transplants, respectively, *p* < 0.001) (Table 1B). Haplo transplants were more likely than UCB to be performed during the study and post-study periods compared to the pre-study period, with OR of 4.6 (95% CI 4.1–5.2) and 20.8 (95% CI 18.0–24.2), respectively, and both with *p* < 0.001. Additionally, haplo transplants were more likely to be performed than UCB during the post-study period compared to the study period with OR 4.5 (95% CI 4.0–5.1), *p* < 0.001 (Table 2).

**Table 1:** Patient characteristics of the Main Cohort by (A) graft type, and (B) three time periods.

	Overall	Haplo	UCB
(A)			
*N*	11,190	8215	2975
Age: median [IQR]	53.7 [38.3, 62.6]	55.3 [40.4, 63.4]	48.6 [33.9, 59.6]
Male: *N* (%)	6443 (57.6)	4864 (59.2)	1579 (53.1)
Race and Ethnicity: *N* (%)			
Non-Hispanic White	6111 (54.6)	4424 (53.9)	1687 (56.7)
Black	1797 (16.1)	1425 (17.3)	372 (12.5)
Hispanic	1944 (17.4)	1433 (17.4)	511 (17.2)
Asian	705 (6.3)	478 (5.8)	227 (7.6)
Other or unknown	633 (5.7)	455 (5.5)	178 (6.0)
Disease: *N* (%)			
Acute leukemia	7381 (66.0)	5185 (63.1)	2196 (73.8)
Chronic leukemia	678 (6.1)	509 (6.2)	169 (5.7)
MDS	1619 (14.5)	1267 (15.4)	352 (11.8)
NHL	979 (8.7)	777 (9.5)	202 (6.8)
HD	134 (1.2)	119 (1.4)	15 (0.5)
MPN	399 (3.6)	358 (4.4)	41 (1.4)

	2010–2012	2013–2018	2019–2022
(B)			
*N*	1434	4915	4841
Graft type, *N* (%)			
Haplo	459 (32.0)	3363 (68.4)	4393 (90.7)
2010	126		
2011	153		
2012	180		
2013		241	
2014		309	
2015		493	
2016		618	
2017		770	
2018		932	
2019			1033
2020			1138
2021			1192
2022			1030
UCB	975 (68.0)	1552 (31.6)	448 (9.3)
2010	303		
2011	343		
2012	329		
2013		339	
2014		310	
2015		276	
2016		234	
2017		206	
2018		187	
2019			176
2020			104
2021			86
2022			82
Age, median [IQR]	49.7 [35.1, 59.5]	53.5 [38.5, 62.4]	55.1 [39.6, 63.5]
Male, *N* (%)	820 (57.2)	2787 (56.7)	2836 (58.6)
Race and Ethnicity, *N* (%)			
Non-Hispanic White	859 (59.9)	2774 (56.4)	2478 (51.2)
Black	243 (16.9)	790 (16.1)	764 (15.8)
Hispanic	195 (13.6)	773 (15.7)	976 (20.2)
Asian	84 (5.9)	294 (6.0)	327 (6.8)
Other or unknown	53 (3.7)	284 (5.8)	296 (6.1)
Disease, *N* (%)			
Acute leukemia	954 (66.5)	3307 (67.3)	3120 (64.4)
Chronic leukemia	141 (9.8)	309 (6.3)	228 (4.7)
MDS	141 (9.8)	692 (14.1)	786 (16.2)
NHL	161 (11.2)	415 (8.4)	403 (8.3)
HD	18 (1.3)	56 (1.1)	60 (1.2)
MPN	19 (1.3)	136 (2.8)	244 (5.0)

*Haplo* haploidentical grafts, *UCB* umbilical cord blood, *IQR* interquartile range, *MDS* myelodysplastic syndrome, *NHL* non-Hodgkin lymphoma, *HD* Hodgkin disease/lymphoma, *MPN* myeloproliferative neoplasm.

**Table 2:** Logistic regression of receipt of haploidentical graft in association with demographic factors by periods in the Main Cohort.

Factor	Description	Odds ratio of receiving Haplo (95% CI)	*P*-value
Time period	During-study vs pre-study	4.6 (4.1, 5.2)	<0.001
	Post-study vs pre-study	20.8 (18.0, 24.2)	<0.001
	Post-study vs during-study	4.5 (4.0, 5.1)	<0.001
Age	≥50 years vs < 50 years	1.74 (1.58, 1.92)	<0.001
Sex	Male vs female	1.32 (1.20, 1.45)	<0.001
Race/ethnicity (compared to non-Hispanic White)	Black	1.74 (1.51, 2.01)	<0.001
	Hispanic	1.03 (0.90, 1.18)	0.665
	Asian	0.75 (0.62, 0.90)	0.002
	Other or unknown	0.87 (0.71, 1.07)	0.193

*Haplo* haploidentical grafts, *CI* confidence interval.

**Fig. 2:**
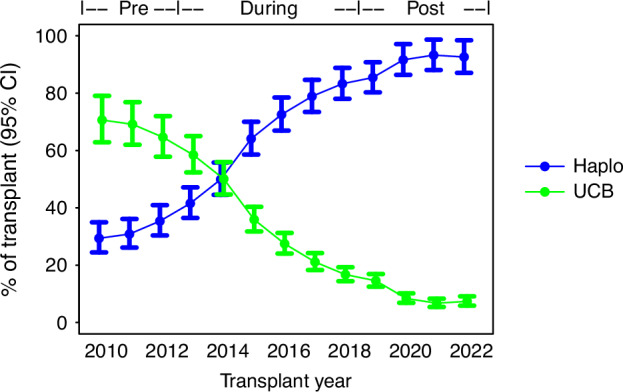
Trend in utilization of haploidentical grafts vs umbilical cord blood over time in the Main Cohort. The three time periods, pre-study (2010–2012), during study (2013–2018), and post-study (2019–2022), were indicated. Haplo haploidentical graft, UCB umbilical cord blood.

To delineate the changes in haplo vs UCB utilization over time, the trend of haplo transplants was also examined by year. We demonstrated the percentage of haplo transplants increased over time while the percentage of UCB transplants diminished over time, with the uptrend of haplo starting around 2013 (Fig. 2). When compared to 2010, the OR of receiving haplo vs UCB for each of the years 2011–2022 increased steadily starting in 2013 (Supplementary Fig. 1)

#### Demographic factors in association with haploidentical graft vs umbilical cord blood utilization

We next analyzed whether the increase in haplo transplants was associated with demographic factors, such as age, sex, and race/ethnicity, and whether the increasing use of haplo varied across different patient populations over time. Among the Main Cohort of 11,190 patients, during 2010–2022, there were 8215 patients receiving haplo transplants, with a median age of 55.3 and 59.2% of whom were male. During this period, there were 2975 patients receiving UCB transplants, with a median age of 48.6 and 53.1% of whom were male (Table 1A). The percentage of patients receiving haplo or UCB transplants who were Hispanic increased over time (13.6%, 15.7%, and 20.2%, respectively for the three time periods). There were also more haplo and UCB transplants for patients with MDS (9.8%, 14.1%, and 16.2%, respectively for the three time periods) and MPN (1.3%, 2.8%, and 5.0%, respectively for the three time periods), reflecting changes in the field across all graft sources (Table 1B).

Through logistic regression by the three time periods, we demonstrated patients 50 years or older were more likely to receive haplo over UCB than younger patients (OR 1.74, with 95% CI 1.58–1.92, *p* < 0.001). Male patients were more likely to receive haplo than female patients (OR 1.32, with 95% CI 1.20–1.45, *p* < 0.001). Compared to NHW recipients, Black recipients were more likely to receive haplo (OR 1.74, with 95% CI 1.51–2.01, *p* < 0.001) while Asian recipients were less likely (OR 0.75 with 95% CI 0.62–0.90, *p* = 0.002) (Table 2). These trends were similarly observed when the analyses were performed by years (Supplementary Table 1).

### Expanded analysis with the extended cohort (61,465 patients)

#### Haploidentical grafts and umbilical cord blood utilization in comparison to other allogeneic donor sources

**Fig. 3:**
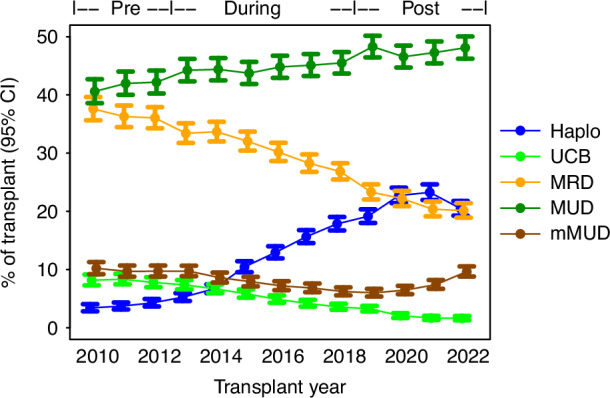
Trend in utilization of haploidentical grafts and umbilical cord blood over time compared to other allogeneic transplants in the Extended Cohort. The three time periods, pre-study (2010–2012), during study (2013–2018), and post-study (2019–2022), were indicated. Haplo haploidentical graft, UCB umbilical cord blood, MRD HLA-identical siblings and HLA-matched other relatives, MUD HLA-matched unrelated donors, mMUD HLA-mismatched unrelated donors.

Haplo and UCB represent important alternative grafts when an HLA-matched donor cannot be found [7, 8]. To assess haplo and UCB utilization compared to other allogeneic grafts (MRD/MUD/mMUD) as a secondary objective of the study, we repeated our analyses using the Extended Cohort (characteristics in Table 3A). We demonstrated the percentage of haplo utilization increased with time (Fig. 3). This trend is also reflected by a steady increase in the number of haplo transplants, with a decline in MRD transplants, and a gradual increase in MUD transplants to a plateau (average number of transplants per year: **pre-study**: 153 haplo, 325 UCB, 1469 MRD, 1671 MUD, 396 mMUD; **during-study**: 561 haplo, 259 UCB, 1473 MRD, 2148 MUD, 371 mMUD; **post-study**: 1098 haplo, 112 UCB, 1107 MRD, 2445 MUD, 378 mMUD) (Table 3B). Of note, mMUD transplants started increasing towards the end of the study period.

**Table 3:** Patient characteristics of the Extended Cohort by (A) graft type, and (B) by three time periods.

	Overall	Haplo	UCB	MRD	MUD	mMUD
(A)						
*N*	61,465	8215	2975	17,674	27,677	4924
Age, median (IQR)	55.7 [42.9, 63.2]	55.3 [40.4, 63.4]	48.6 [33.9, 59.6]	54.6 [43.1, 61.6]	57.5 [45.2, 64.5]	54.4 [40.7, 62.7]
Male, *N* (%)	35,266 (57.0)	4864 (59.2)	1579 (53.1)	10,144 (57.4)	15,742 (56.9)	2702 (54.9)
Non-Hispanic White, *N* (%)	45,492 (73.5)	4424 (53.9)	1687 (56.7)	12,389 (70.1)	23,581 (85.2)	3158 (64.1)
Black, *N* (%)	3999 (6.5)	1425 (17.3)	372 (12.5)	1033 (5.8)	589 (2.1)	535 (10.9)
Hispanic, *N* (%)	7091 (11.5)	1433 (17.4)	511 (17.2)	2612 (14.8)	1704 (6.2)	757 (15.4)
Asian, *N* (%)	2485 (4.0)	478 (5.8)	227 (7.6)	844 (4.8)	696 (2.5)	219 (4.4)
Other or Unknown, *N* (%)	2813 (4.5)	455 (5.5)	178 (6.0)	796 (4.5)	1107 (4.0)	255 (5.2)

	Overall	2010–2012	2013–2018	2019–2022
(B)				
*N*	61,465	12,041	28,866	20,558
Average *N* per year	4728	4013	4811	5140
Age, median (IQR)	55.72 (42.91, 63.24)	53.41 (41.44, 60.96)	55.71 (43.23, 63.17)	57.31 (43.48, 64.50)
Male, *N* (%)	35,031 (57.0)	6958 (57.8)	16,417 (56.9)	11,656 (56.7)
Non-Hispanic White, *N* (%)	45,239 (73.6)	9520 (79.1)	21,467 (74.4)	14,252 (69.3)
Black, *N* (%)	3954 (6.4)	720 (6.0)	1802 (6.2)	1432 (7.0)
Hispanic, *N* (%)	7017 (11.4)	1127 (9.4)	3122 (10.8)	2768 (13.5)
Asian, *N* (%)	2464 (4.0)	419 (3.5)	1114 (3.9)	931 (4.5)
Other/Unknown, *N* (%)	2791 (4.5)	255 (2.1)	1361 (4.7)	1175 (5.7)
Haplo	8215 (13.4)	459 (3.8)	3363 (11.7)	4393 (21.4)
UCB	2975 (4.8)	975 (8.1)	1552 (5.4)	448 (2.2)
MRD	17,674 (28.8)	4408 (36.6)	8840 (30.6)	4426 (21.5)
MUD	27,677 (45.0)	5012 (41.6)	12,887 (44.6)	9778 (47.6)
mMUD	4924 (8.0)	1187 (9.9)	2224 (7.7)	1513 (7.4)

*Haplo* haploidentical grafts, *UCB* umbilical cord blood, *MRD* HLA-identical siblings and HLA-matched other relatives, *MUD* HLA-matched unrelated donors, *mMUD* HLA-mismatched unrelated donors, *IQR* interquartile range.

#### Haploidentical grafts and umbilical cord blood as alternative donor sources for racial/ethnic groups with limited donor availability

Haplo and UCB availability improved access to transplant for many racial/ethnic groups. Therefore, we assessed the association between race/ethnicity and haplo and UCB utilization in our Extended Cohort. We found among all graft sources during 2010–2022, for Black, Hispanic, and Asian patients, haplo and UCB grafts represent significantly higher proportions compared to NHW patients. Additionally, haplo is the major donor source for Black patients (reported here: number of transplants [percentage of the graft source out of all sources for each racial/ethnic group]. **NHW**: haplo: 4424 [9.8%], UCB: 1687 [3.7%], MRD: 12,389 [27.4%], MUD: 23,581 [52.1%], mMUD: 3158 [7.0%]; **Black**: haplo: 1425 [36.0%], UCB: 372 [9.4%], MRD: 1033 [26.1%], MUD: 589 [14.9%], mMUD: 535 [13.5%]; **Hispanic**: haplo: 1433 [20.4%], UCB: 511 [7.3%], MRD: 2612 [37.2%], MUD: 1704 [24.3%], mMUD: 757 [10.8%]; **Asian**: haplo: 478 [19.4%], UCB: 227 [9.2%], MRD: 844 [34.3%], MUD: 696 [28.3%], mMUD: 219 [8.9%]) (Table 3A).

More importantly, there were increases in transplants from any graft source for all patients, regardless of race/ethnicity (average number of transplants of any graft per year: **pre-study**: NHW: 3173, Black: 240, Hispanic: 376, Asian: 140; **during-study**: NHW: 3578, Black: 300, Hispanic: 520, Asian: 186; **post-study**: NHW: 3563, Black: 358, Hispanic: 692, Asian: 233) (Table 3B). These increases were driven particularly from mismatched grafts (mMUD, haplo, UCB) for all racial/ethnic groups and were not paralleled by matched grafts (MRD and MUD) (Supplementary Fig. 2).

Lastly, using multinomial logistic regression, with MUD as the reference group given its major representation, we showed haplo and UCB are much more likely to be utilized in non-White patients, especially Black patients (RRR 12.73, *p* < 0.001 for haplo, and RRR 8.29, *p* < 0.001 for UCB, respectively), with an increasing trend over time for haplo and a declining trend for UCB over time. Similarly to haplo and UCB, mMUD is more frequently used in non-White race/ethnicity (Table 4).

**Table 4:** Relative risk ratios of multinomial logistic regression model for the type of transplant.

	Year of Transplant from 2010	Male vs Female	Black vs White	Hispanic vs White	Asian vs White	Other or unknown vs White	Age ≥ 50 years vs < 50 years
Haplo vs MUD	1.15 (1.14, 1.16)	1.19 (1.13, 1.26)	12.73 (11.49, 14.12)	4.05 (3.74, 4.38)	3.39 (3.00, 3.83)	1.91 (1.70, 2.14)	0.97 (0.92, 1.02)**
UCB vs MUD	0.85 (0.84, 0.86)	0.91 (0.84, 0.98)*	8.29 (7.19, 9.55)	4.08 (3.64, 4.58)	4.58 (3.90, 5.38)	2.64 (2.23, 3.13)	0.59 (0.55, 0.64)
MRD vs MUD	0.92 (0.92, 0.93)	1.04 (1.00, 1.08)*	3.39 (3.06, 3.77)	3.10 (2.90, 3.31)	2.43 (2.19, 2.70)	1.52 (1.38, 1.67)	0.99 (0.95, 1.03)**
mMUD vs MUD	0.95 (0.94, 0.96)	0.96 (0.90, 1.02)**	6.71 (5.93, 7.60)	3.35 (3.04, 3.68)	2.38 (2.04, 2.79)	1.82 (1.58, 2.09)	0.90 (0.84, 0.96)

*Haplo* haploidentical grafts, *UCB* umbilical cord blood, *MRD* HLA-identical siblings and HLA-matched other relatives, *MUD* HLA-matched unrelated donors, *mMUD* HLA-mismatched unrelated donors.

*p* all <0.001, except for cells with *p < 0.05 and **p > 0.05.

## Discussion

Our real-world study examines the change in graft source selection, before, during, and after the publication of the randomized trial CTN-1101 comparing outcomes of reduced-intensity haplo vs UCB [3, 4]. In this large observational study, we have analyzed 11,190 patients who underwent haplo or UCB transplants between 2010 and 2022 at 246 U.S. transplant centers. We demonstrated that the trend of utilizing haplo as a stem cell source has been significantly increasing since 2013. As the CTN-1101 trial started to enroll patients in June 2012 [4], these findings suggest practice change toward haplo utilization had begun before the CTN-1101 trial’s publication. One of the explanations for this practice change could be the increasing use of post-transplant cyclophosphamide (PTCy) for graft-versus-host disease (GVHD) prophylaxis for haplo that increased haplo utilization [9]. This is evidenced by the fact that, after the report in 2008 by Luznik et al. [10], PTCy as GVHD prophylaxis started to be increasingly utilized around 2013 and was consistently used in ≥85% of haplo transplants after 2015 [9]. Our data also showed a significant increase in haplo transplants after the CTN-1101’s findings were first presented in February 2020 (haplo constituting 91% of the total haplo and UCB transplants during the post-study period vs 68% during the study, *p* < 0.001; OR of 4.5, *p* < 0.001 post-study vs during-study), suggesting presentation and publication of the trial’s results may have impacted the trend of already increasing haplo utilization, further reinforcing this clinical practice.

The worldwide donor registry pool is comprised of more than 42 million donors, with the majority of European ancestry [11]. The probability for a patient with European ancestry to find an HLA-matched unrelated donor (MUD) is 79%, whereas the probabilities of finding a MUD for Black, Asian, and Hispanic patients are as low as 29%, 50%, and 48%, respectively [12]. The use of allogeneic HCT for acute myeloid leukemia, MDS, and acute lymphoblastic leukemia was lower during 2009–2018 for Black patients compared to NHW patients [13]. The need for alternative donor grafts becomes even more pressing as the demographics in the US and Europe are changing, with an increasing number of patients of Asian and Hispanic backgrounds and of mixed ancestry [14]. Recent data from the CTN 1702 trial suggest patients who are unlikely to have a matched donor should proceed with HCT using alternative donor grafts [15]. UCB has recently been demonstrated to have improved overall survival over time in all racial/ethnic groups [16]. Moreover, haplo and UCB transplants convey similar relapse rates, overall survival, and disease-free survival for Black patients [17, 18]. Therefore, both UCB and haplo offer an alternative transplant option for patients of race/ethnicity with limited donor availability. Through logistic regression analyses, we have shown over the years, Black patients were more likely than NHW patients to receive haplo than UCB (with the Primary Cohort analysis), and to receive haplo compared to matched related or unrelated donors, or mismatched unrelated donors (with the Extended Cohort analysis). Similarly, Asian and Hispanic patients were more likely than NHW patients to receive haplo with the Extended Cohort analysis. These findings are encouraging, as they suggest that access to HCT is expanded through alternative graft sources such as haplo and UCB. More importantly, through our Extended Cohort analysis, we have shown over time, the number of transplants from any graft source increases for Black, Hispanic, and Asian patients, further painting a promising outlook for access to HCT for all patients.

In this study, we included only U.S. centers to be consistent with the CTN-1101’s inclusion criteria [4]. Worldwide, in Japan, haplo transplant with PTCy prophylaxis and UCB interestingly have comparable two-year overall survival and relapse free survival, but haplo transplant with PTCy prophylaxis did have a lower cumulative incidence of non-relapse mortality than UCB [19]. In Europe, while haplo utilization between 2013 and 2022 mirrored what we observed in U.S. centers, UCB utilization were mostly plateauing between 2018 and 2022 (whereas UCB transplants were declining in the U.S. during this period). Notably, unrelated donor transplants in Europe were increasing dramatically between 2010 and 2022, whereas our analysis showed unrelated donor transplants in the U.S. were stable to only slightly increased [20].

While our study revealed several important findings, there were also limitations. The registry data only represent the final utilized donor for those that had an HCT. Also, the lack of body mass index in our dataset did not allow for accounting this important factor which could influence a clinician’s decision in graft choice [21]. The lack of zip codes and insurance/payer status in our dataset did not allow for potential association between socioeconomic status and graft utilization, therefore limiting the ability to assess structural disparities in access to transplant. Furthermore, data regarding referral pattern and center-specific characteristics were not available with our de-identified dataset, and we were unable to control for supportive care improvements. However, Fingerson et al. have shown that practice patterns at high-volume haplo transplant centers were not uniform [22], which suggests center-specific factors were less likely to be underlying the observed trends in this study. Notably within our dataset, prior to the CTN-1101, there were 120 centers performing UCB transplants. This number decreased to 103 centers in the post-study period, corroborating UCB transplants decreasing with time.

Additionally, recent studies have shown PTCy significantly improves transplant outcomes and bridges the outcome gaps between MUD and mMUD independent of a patient’s ancestry [23, 24]. Our study only extends to 2022, and therefore mMUD was not the focus of our analysis. However, in the PTCy era, mMUD is another promising alternative graft source for patient of diverse race/ethnicity.

In conclusion, we have shown the CTN-1101 trial did have an impact on the utilization of haplo transplant over UCB after its publication and dissemination. Furthermore, our findings demonstrate HLA-mismatched donors, including haplo, UCB, and mMUD, are recognized as vital alternative graft sources, utilized to allow patients of all backgrounds to have access to HCT. In our future studies, we plan to analyze socioeconomic and other factors that could influence the choice of graft source. Additionally, we plan to investigate the graft source options and choices with PTCy-based GVHD prophylaxis.

## Supplementary information

Supplemental Information

## Data Availability

The materials described in the manuscript, including all relevant raw data, will be freely available to any researcher wishing to use them for non-commercial purposes, without breaching participant confidentiality.
