# Supplementary material for: Real-world graft utilization after CTN-1101: a registry-based analysis of haploidentical graft versus umbilical cord blood trends
Source: Bone Marrow Transplant. 2025 Aug 18;60(10):1369–76. doi: 10.1038/s41409-025-02694-z (PMC12568632; doi:10.1038/s41409-025-02694-z)

**SUPPLEMENTAL INFORMATION**

**Supplemental Table 1:** **Logistic regression of receipt of haploidentical graft in association with demographic factors by years in the Main Cohort**. Haplo: haploidentical grafts; CI: confidence interval

| **Factor** | **Description** | **Odds Ratio of Receiving Haplo (95% CI)** | **P-value** |
| --- | --- | --- | --- |
| **Age** | Age ≥50 years vs <50 years | 1.71 (1.55, 1.89) | <0.001 |
| **Sex** | Male vs Female | 1.32 (1.20, 1.45) | <0.001 |
| **Race/ethnicity (compared to non-Hispanic White patients)** | Black | 2.70 (1.98, 3.67) | <0.001 |
|  | Hispanic | 0.72 (0.51, 1.02) | 0.069 |
|  | Asian | 0.77 (0.46, 1.39) | 0.302 |
|  | Other or unknown | 0.80 (0.45, 1.39) | 0.443 |
| **Year of Transplant** | Year of transplant minus 2009 as continues variable | 1.41 (1.39, 1.44) | <0.001 |
| **Interaction of Transplant Year and Race/Ethnicity (compared to non-Hispanic White patients)** | Black | 0.94 (0.90, 0.98) | 0.003 |
|  | Hispanic | 1.04 (0.99, 1.08) | 0.099 |
|  | Asian | 0.99 (0.93, 1.05) | 0.705 |
|  | Other or unknown | 1.00 (0.94, 1.08) | 0.911 |

**Supplemental Figure 1:** **The likelihood of receiving haploidentical grafts vs umbilical cord blood during each year of the period 2011-2022 compared to 2010 in the Main Cohort.** p<0.05 for all years starting in 2013 until 2022, when compared to 2010.


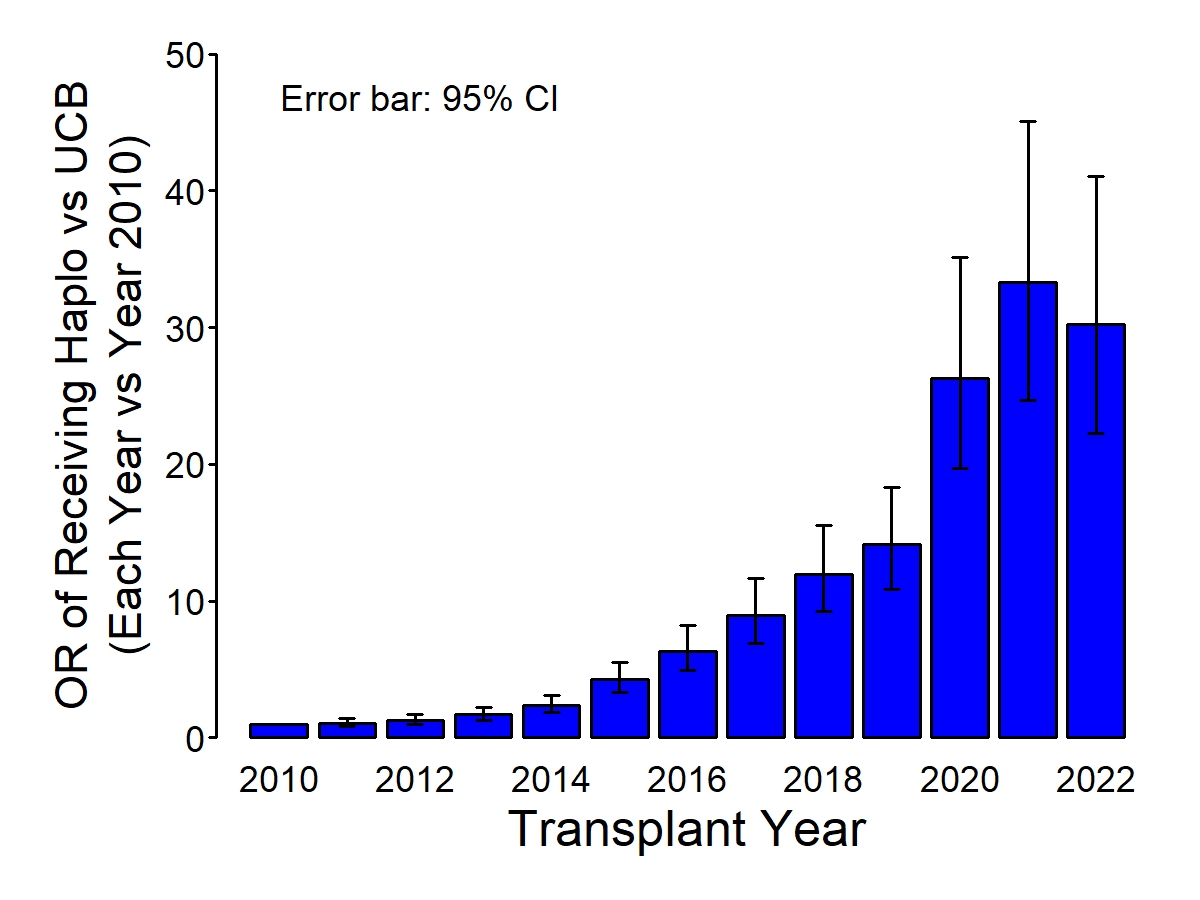


**Supplemental Figure 2**: **Trend in utilization of all graft sources in diverse race/ethnicity in the Extended Cohort.**


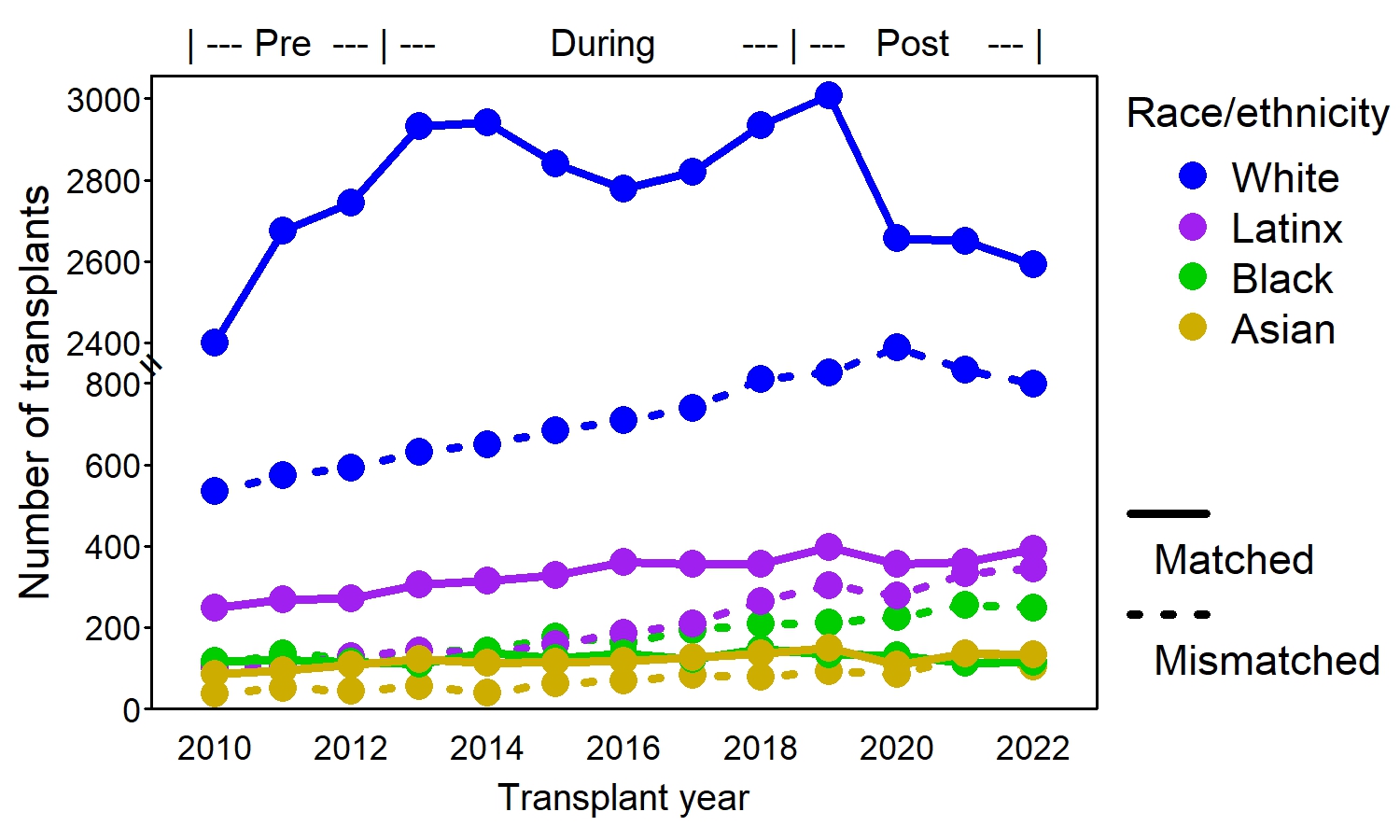

Supplement: Supplementary file 1 — Supplemental Information [file 41409_2025_2694_MOESM1_ESM.docx]
